# Ultrastructural study of vitellogenesis and oogenesis of *Crepidostomum metoecus* (Digenea, Allocreadiidae), intestinal parasite of *Salmo trutta* (Pisces, Teleostei)

**DOI:** 10.1051/parasite/2016057

**Published:** 2016-11-15

**Authors:** Samuel Greani, Yann Quilichini, Bernard Marchand

**Affiliations:** 1 University of Corsica, CNRS, UMR 6134 – SPE, Parasites and Mediterranean Ecosystems Laboratory 20250 Corte Corsica France

**Keywords:** Oogenesis, Vitellogenesis, *Crepidostomum*, Digenea

## Abstract

We describe the vitellogenesis and oogenesis of *Crepidostomum metoecus* from *Salmo trutta* collected in Corsica. This is the first study conducted in the Allocreadiidae family. The maturation of *C. metoecus* vitellocytes comprises four different stages depending on organelle content. The follicular vitellarium is surrounded by a basal lamina. Vitellocytes are randomly distributed into the vitellarium, although fully mature vitellocytes are found in the center of the follicle. During maturation, the nucleo-cytoplasmic ratio decreases, whereas synthetic activity increases. Fully mature vitellocytes are filled with β-glycogen particles and shell globule clusters. Compared to other trematodes studied, *C. metoecus* possesses a large amount of nutritive reserves for the developing embryo and high quantities of material for the developing shell. Oocyte maturation takes place in four stages: oogonia, primary oocytes, developing oocytes, and mature oocytes. Developing oocytes enter the zygotene-pachytene stage of the first meiotic division recognizable by the presence of synaptonemal complexes in the nucleoplasm. The low protein composition of mature oocytes associated with the large nutrient content of vitellocytes of *C. metoecus* enables us to consider that oocytes do not take part of the nutrition of the future embryo of the miracidium. A cytochemical test (Thiéry method) allowed us to detect the presence of polysaccharides and glycogen during maturation of these two cell types.

## Abstract (fr)

Nous décrivons la vitellogenèse de *Crepidostomum metoecus* (de *Salmo trutta* récolté en Corse). Cette étude est la première menée dans la famille Allocreadiidae. La maturation des vitellocytes de *C. metoecus* comprend quatre stades différents, en fonction des organites qu’ils contiennent. Le vitellarium folliculaire est entouré par une lame basale. Les vitellocytes sont distribués au hasard dans le vitellarium, cependant les vitellocytes pleinement mûrs se trouvent dans le centre du follicule. Au cours de la maturation le rapport nucléo-cytoplasmique diminue, tandis que l’activité de synthèse augmente. Les vitellocytes mûrs contiennent des particules de β-glycogène et des grappes de globules pour la formation de la coquille. Par rapport à d’autres trématodes étudiés, *C. metoecus* possède une grande quantité de réserves nutritives pour le développement de l’embryon, et beaucoup de matériel pour le développement de la coquille. La maturation des ovocytes se déroule en quatre étapes: ovogonies, ovocytes primaires, ovocytes en développement, et ovocytes mûrs. Les ovocytes en développement entrent dans la phase zygotène-pachytène de la première division méiotique, reconnaissable par la présence de complexes synaptonémaux dans le nucléoplasme. La faible composition en protéines des ovocytes mûrs associée à la grande teneur en éléments nutritifs des vitellocytes de *C. metoecus*, nous permettent de considérer que les ovocytes ne participent pas à la nutrition du futur embryon du miracidium. Un test cytochimique (méthode de Thiéry) nous a permis de localiser la présence de polysaccharides et de glycogène pendant ces deux maturations cellulaires.

## Introduction

The absence of fossils is a feature in Platyhelminthes, especially parasites. Therefore, to understand the relationships between species and their phylogeny, it is possible to research extant taxa, and include morphological and ultrastructural data, and more recently molecular analysis findings. Although the number of ultrastructural studies of vitellogenesis in digeneans has increased, several species (or families) have not been significantly examined to date. In fact, among the 18,000 digenean species [3, 10] that have been described, fewer than 20 were studied for their vitellogenesis [7, 8, 12–15, 21–24, 30, 32, 37, 42, 45, 46]. *Crepidostomum metoecus* (Braun, 1900) is already listed in several studies as a parasite of brown trout in European countries [39]. According to Quilichini et al. (2007), the wide geographical distribution has led to diversity of intermediate hosts [39]. The vitelline cells provide the material necessary for the formation of the eggshell and the essential nutrient material for the development of the future embryo. The oogenesis of trematodes has been the subject of several studies by light and electron microscope [2, 4, 6, 16, 18, 20, 25, 29, 31, 33, 40, 43, 44, 48, 49, 50–53]. The female reproductive system of Platyhelminthes shows great morphological variability with significant differences in anatomical organization and cell structure between taxa. Platyhelminthes have been subdivided into two levels of organization, according to the female reproductive system. The Archoophora possess homocellular female gonads consisting of only germaria with oocytes which produce both yolk and eggshell forming precursors. The Neoophora are characterized by heterocellular female gonads composed of an ovary and vitelline glands. The neoophoran digenean *Crepidostomum metoecus* belongs to the Allocreadiidae family (Looss, 1902). The Allocreadiidae are relatively small digeneans that are parasites of the digestive system of teleosts, and occasionally snakes, salamanders, and frogs. The present study shows, for the first time, the ultrastructure of the female gonads of an Allocreadiidae. The aim of this study was to describe the ultrastructural characteristics of oocytes and vitellocytes during their differentiation.

## Materials and methods

Adult specimens of *Crepidostomum metoecus* were collected live from the intestine of naturally infected *Salmo trutta* (Linnaeus, 1758) collected in Corsica. Worms were removed from their hosts, fixed in cold (4 °C) 2.5% glutaraldehyde in 0.1 M sodium cacodylate buffer at pH 7.2, rinsed in 0.1 M sodium cacodylate buffer at pH 7.2, postfixed in cold (4 °C) 1% osmium tetroxide in the same buffer for 1 h, dehydrated in ethanol and propylene oxide, embedded in Spurr, and polymerized at 60 °C for 24 h. Ultrathin sections (60–90 nm) of the worms, at the level of the ovary or vitelline follicles, were cut on an ultramicrotome (PowerTome PC, RMC Boeckeler). Sections were placed on 300- and 200-mesh copper and gold grids. Sections on copper grids were stained with uranyl acetate and lead citrate [41]. Sections on gold grids were stained with periodic acid, thiocarbohydrazide, and silver proteinate [47]. This technique was used to detect glycogen. Sections were examined on a Hitachi H-7650 transmission electron microscope, operating at an accelerating voltage of 80 kV, in the “*Service d’Étude et de Recherche en Microscopie Électronique*” of the University of Corsica (Corte, France).

## Results

### Vitellogenesis

**Figures 1–4.**
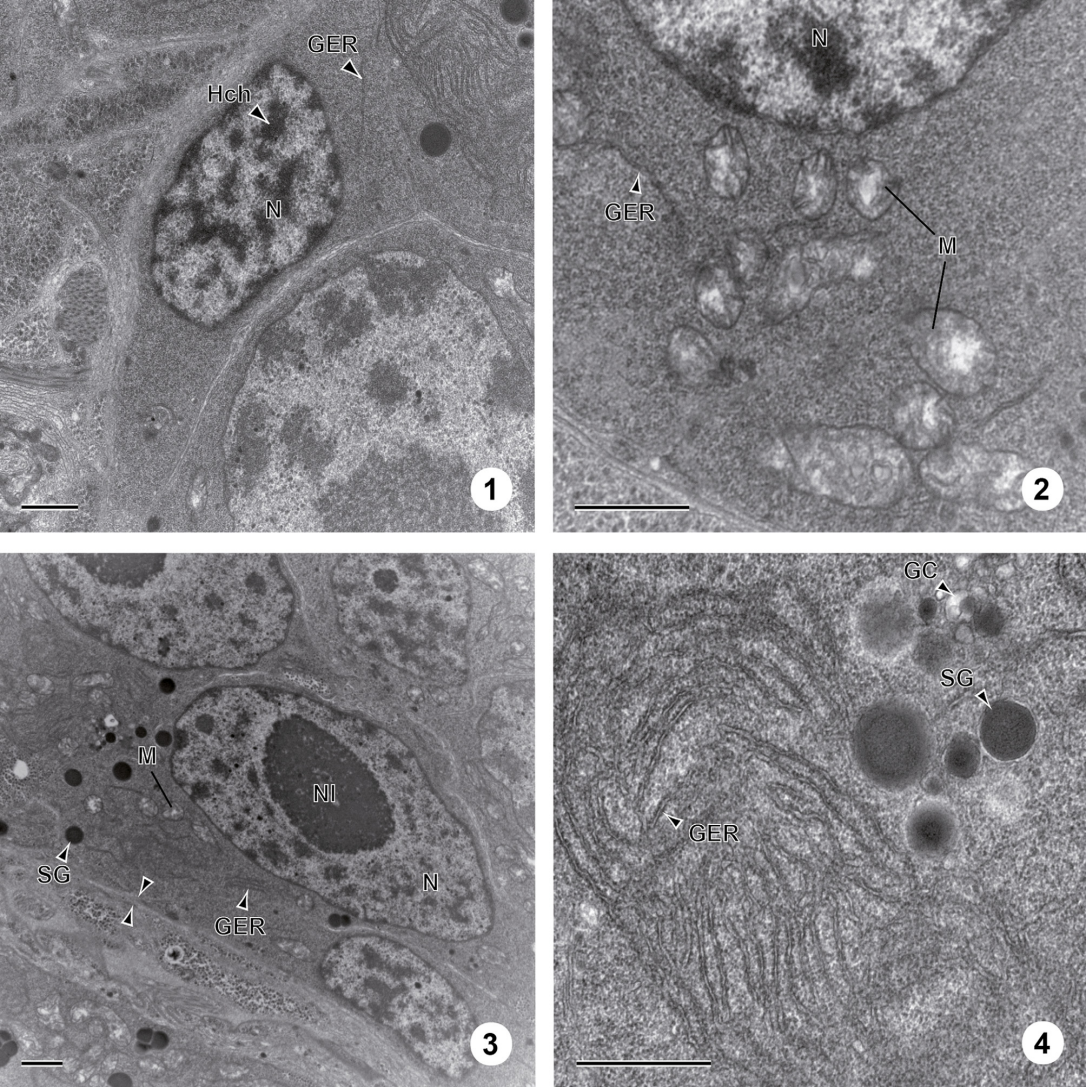
Stages 1 and 2 of vitellogenesis in *C. metoecus*. (1) An immature vitellocyte (Stage 1) at the periphery of the follicle. The nucleus is filled with heterochromatin and the cell possesses a high nucleo-cytoplasmic ratio. Scale bar = 1 μm. (2) Immature vitellocyte at first stage of maturation filled with ribosomes, mitochondria, and few endoplasmic reticulum saccules. Scale bar = 1 μm. (3) Electron micrograph of second stage of vitelline cells’ maturation (arrowheads: basal lamina). Scale bar = 1 μm. (4) Part of the cytoplasm of a cell at second stage of maturation showing Golgi complexes and granular endoplasmic reticulum. Scale bar = 1 μm. GC: golgi complex; GER: granular endoplasmic reticulum; Hch: heterochromatin; M: mitochondrion; N: nucleus; Nl: nucleolus; SG: shell globule.

**Figures 5–8.**
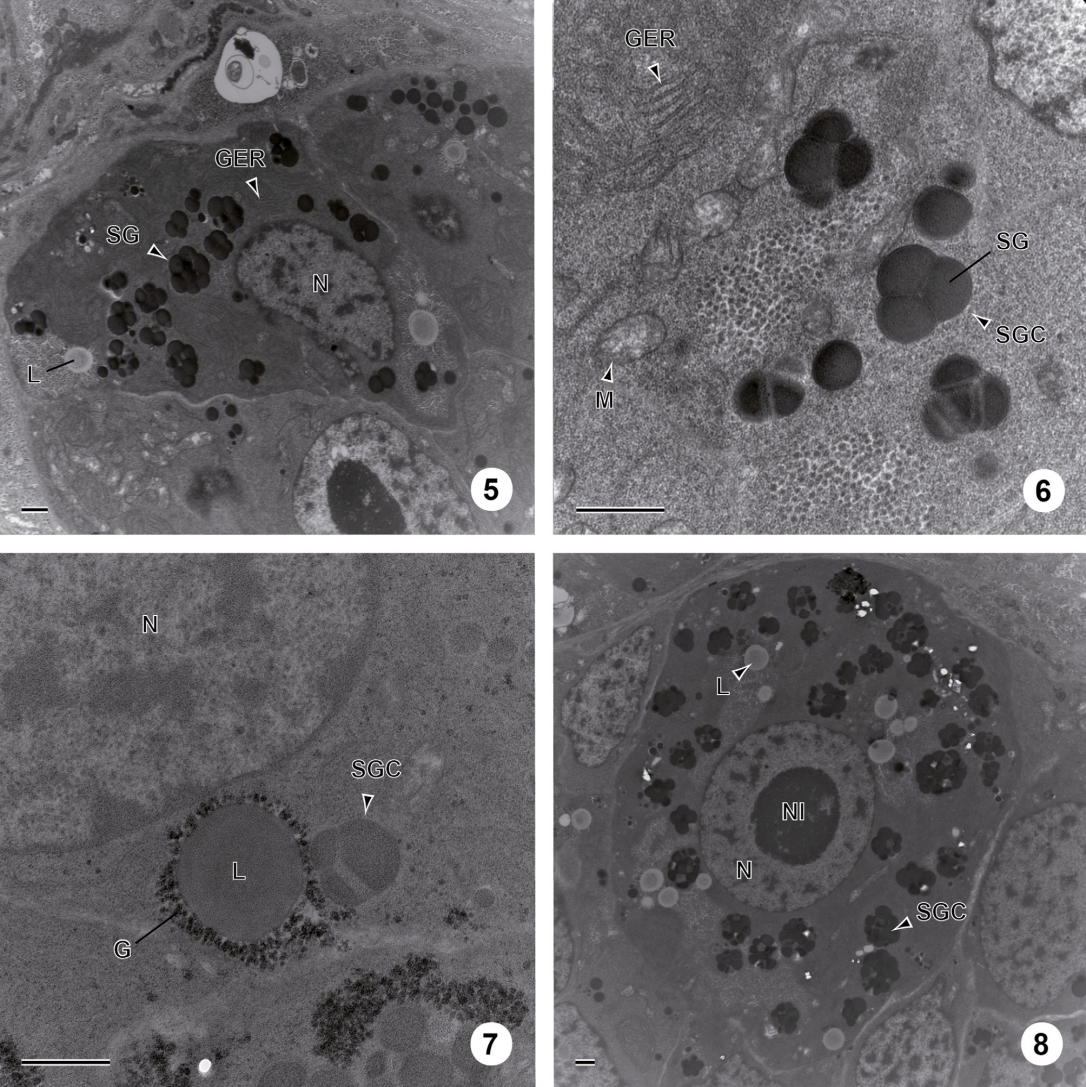
Stages 3 and 4 of vitellogenesis in *C. metoecus*. (5) General observation of a vitelline cell at the third stage of maturation. Scale bar = 1 μm. (6) The third stage of vitellocyte maturation showing the coalescence of single shell globules into a cluster surrounded by a membrane. Scale bar = 1 μm. (7) Cytoplasm of a vitelline cell at the third stage of maturation containing shell globule cluster and saturated lipid droplets, surrounded by glycogen granules. Stained according to the Thiéry method. Scale bar = 1 μm. (8) Electron micrograph of a vitelline cell at the fourth stage of maturation filled with shell globule clusters and lipid droplets. Scale bar = 1 μm. G: glycogen particle; GER: granular endoplasmic reticulum; L: lipid droplet; M: mitochondrion; N: nucleus; Nl: nucleolus; SG: shell globule; SGC: shell globule cluster.

**Figure 9–12.**
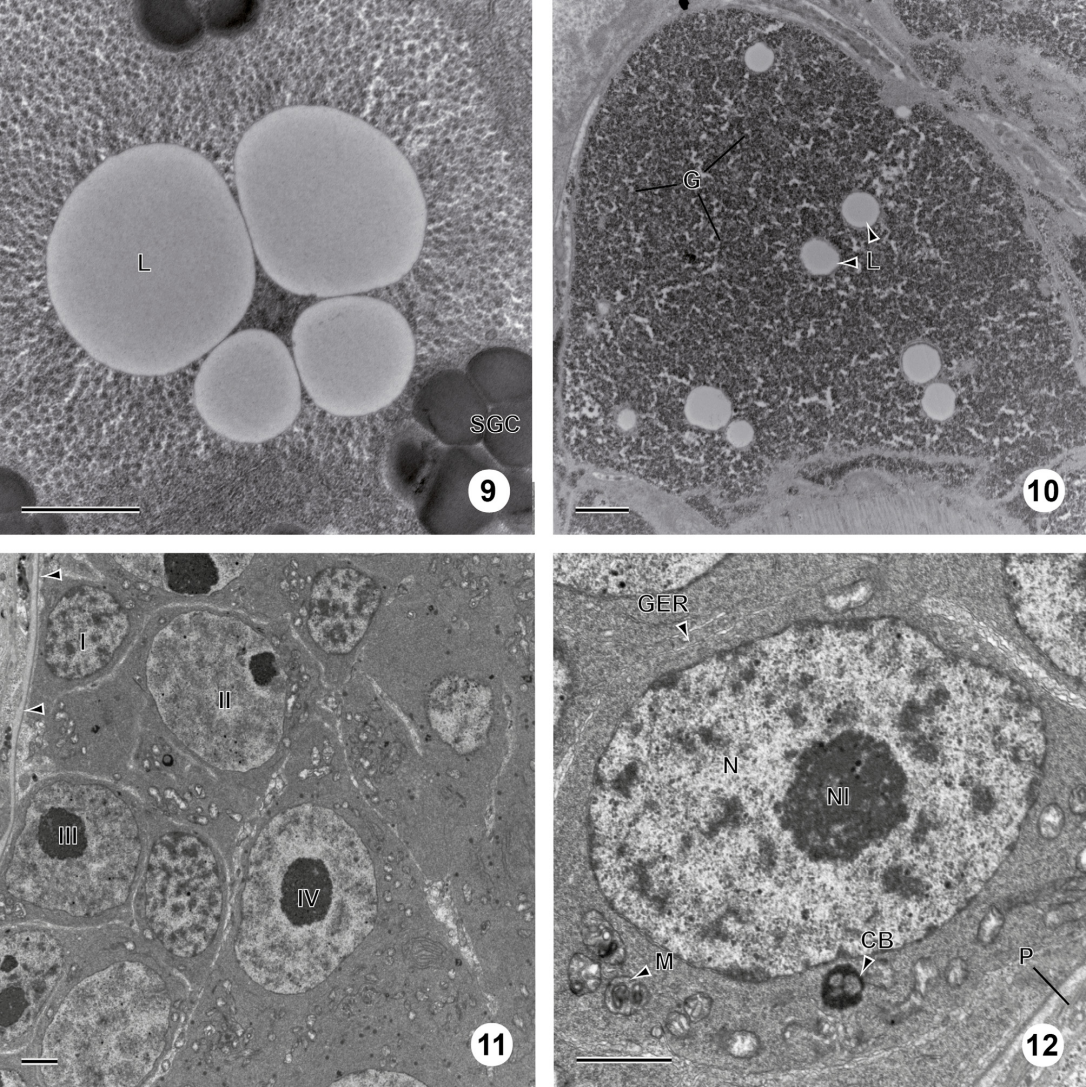
Stage 4 of vitellogenesis and primary oocyte stage of oogenesis in *C. metoecus*. (9) A group of saturated lipid droplets, surrounded by glycogen granules, and a few shell globule clusters. Scale bar = 1 μm. (10) A part of the cytoplasm of a vitelline cell at the fourth stage of maturation. Note the abundance of glycogen granules around lipid droplets. Stained according to the Thiéry method. Scale bar = 1 μm. (11) Electron micrograph cross-section of ovary (arrowheads: basal lamina). Germ cells of the four stages of maturation are observed (I–IV). Scale bar = 2 μm. (12) Primary oocyte showing a small amount of cytoplasm, filled with free ribosomes, mitochondria, few granular endoplasmic reticula, and a chromatoid body. Scale bar = 1.5 μm. CB: chromatoid body; G: glycogen particle; GER: granular endoplasmic reticulum; L: lipid droplet; M: mitochondrion; N: nucleus; Nl: nucleolus; P: parenchyma; SGC: shell globule cluster.

The vitelline glands of *C. metoecus* contain vitellocytes at various stages of development, with younger cells localized in the periphery of the vitelline lobes. One cell type is observed and there are no cytoplasmic extensions between vitellocytes. Follicular vitellarium is surrounded by a basal lamina (Figs. 1, 3). Vitellocyte maturation is divided into four main stages. At the first stage, vitellocytes are undifferentiated cells showing a high nucleo-cytoplasmic ratio (Figs. 1, 15) and measure about 6 μm in diameter. The cytoplasm is mainly filled with free ribosomes, some mitochondria, and scarce endoplasmic reticulum sacculi (Fig. 2). Some heterochromatin is present in the nucleoplasm. At the second stage, the nucleo-cytoplasmic ratio decreases (Figs. 3, 15), and Golgi complexes are evident in the cytoplasm (Fig. 4). At this stage, single shell inclusions coalesce to form clusters of electron-dense globules delimited by a membrane and measure about 10 μm in diameter. The nucleus often contains a large nucleolus (Fig. 3). At the third stage, single shell globules coalesce into clusters (Figs. 5, 15) and vitellocytes measure about 14 μm in diameter. There are less mitochondria and reticulum endoplasmic sacculi (Fig. 6). Some small lipid droplets were seen in the cytoplasm, and glycogen particles were detected according to the Thiéry method (Fig. 7). At the last stage, there are many shell globule clusters in the cytoplasm (>30 per cross-section), measuring about 19 μm in diameter. The cytoplasm also contains some mitochondria, and scarce endoplasmic reticulum sacculi at the periphery of the cell (Fig. 8). Mature vitellocytes of *Crepidostomum metoecus* possess a high amount of saturated lipid content (>10 lipid droplets per cell cross-section) (Figs. 8, 9) and many glycogen particles (Figs. 10, 15).

**Figure 13 and 14.**
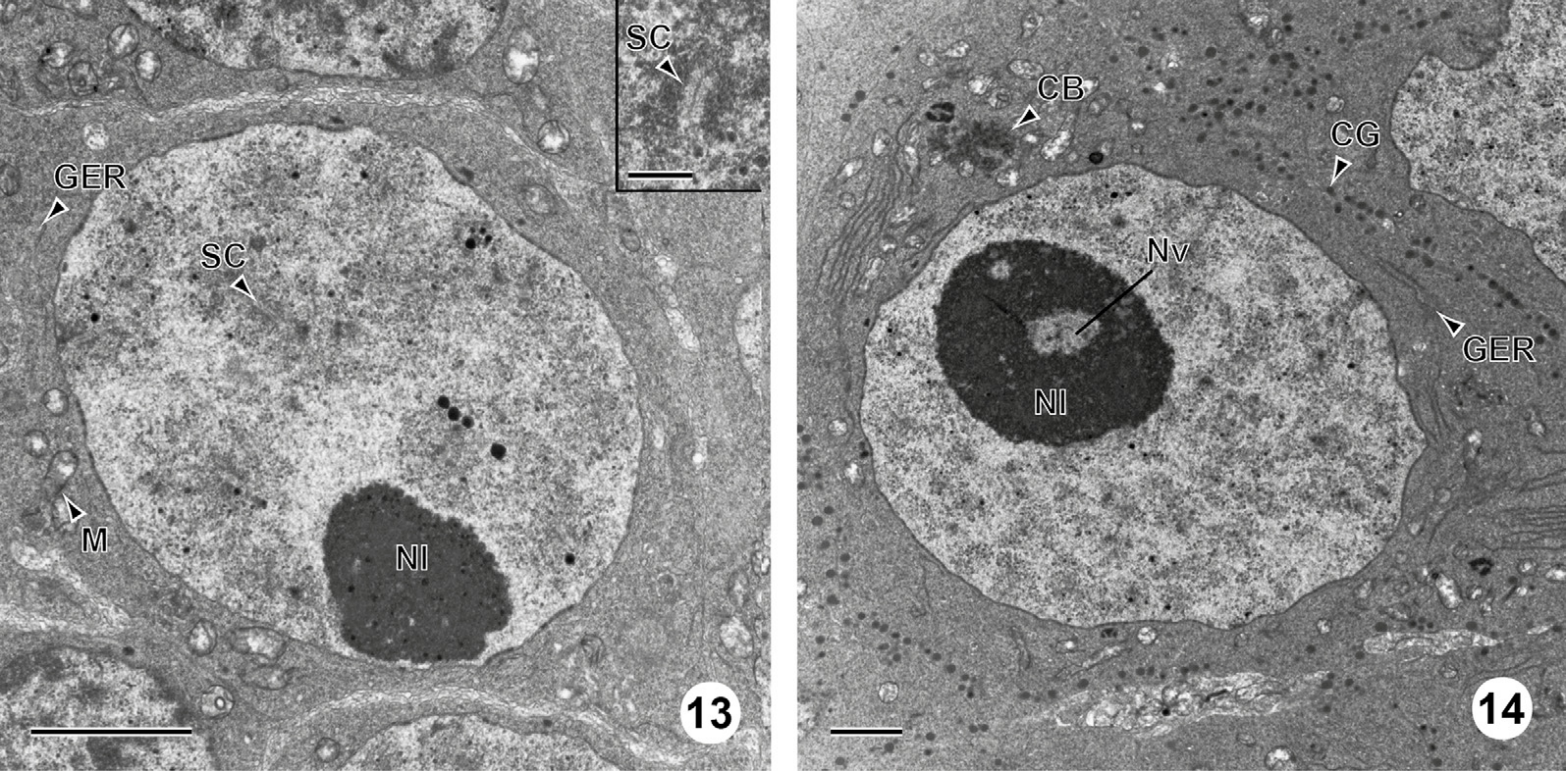
Developing and mature oocytes in *C. metoecus*. (13) General observation of a developing oocyte. Note the presence of synaptonemal complexes indicating the zygotene-pachytene stage of the first meiotic division. Inset: detail of a synaptonemal complex. Scale bar = 2 μm. (14) General observation of a fully mature germ cell, with a low nucleo-cytoplasmic ratio. Cortical granules are at the periphery of the cell, and a chromatoid body is surrounded by clusters of mitochondria in the cytoplasm. Nuclear vacuoles are observed in the nucleolus. Scale bar = 2 μm. CB: chromatoid body; CG: cortical granule; GER: granular endoplasmic reticulum; M: mitochondrion; Nl: nucleolus; Nv: nuclear vacuole; SC: synaptonemal complex.

### Oogenesis

The ovary is surrounded by a basal lamina (Fig. 11). It contains germ cells at various development stages that are closely packed, with younger cells generally localized in the periphery of the ovarian lobes (Fig. 11). Few organelles are observed between cells in the ovary. Four stages are observed during oogenesis in *C. metoecus*: oogonia, primary oocytes, developing oocytes, and mature oocytes. Oogonia are typically undifferentiated cells, showing a high nucleo-cytoplasmic ratio, measure about 5 μm in diameter and have a roundish shape. The scant cytoplasm is filled with free ribosomes and contains few mitochondria (Figs. 11, 16); no nucleoli are present in the nucleus, only heterochromatin. Primary oocytes possess a higher nucleo-cytoplasmic ratio (Figs. 11, 12, 16) and measure about 8 μm in diameter. The cytoplasm contains chromatoid bodies in the perinuclear region and is filled with free ribosomes, mitochondria, and few granular endoplasmic reticula. The nucleus often shows a nucleolus at this stage. Developing oocytes (Figs. 11, 13, 16) enter in the zygotene-pachytene stage of the first meiotic division, as evidenced by the appearance of synaptonemal complexes in the nucleus (Fig. 13 inset) and measure about 11 μm in diameter. There may be up to eight synaptonemal complexes in a nucleus (by transversal section). The cytoplasm contains less mitochondrion but more granular endoplasmic reticulum. The mature oocytes are located in the central region of the ovary (Figs. 11, 14, 16) and possess elongated cytoplasmic extensions. These cells often show a triangular shape and measure about 18 μm in diameter. In all mature oocytes, a chromatoid body (2 μm of diameter) is present in the cytoplasm, near the granular endoplasmic reticulum and surrounded by mitochondria. Cortical granules are in a monolayer close to the periphery of the cell (approximately 50 per cell). The granular endoplasmic reticulum often takes a parallel shape, and mitochondria are clustered at a pole of the cell.

**Figure 15.**
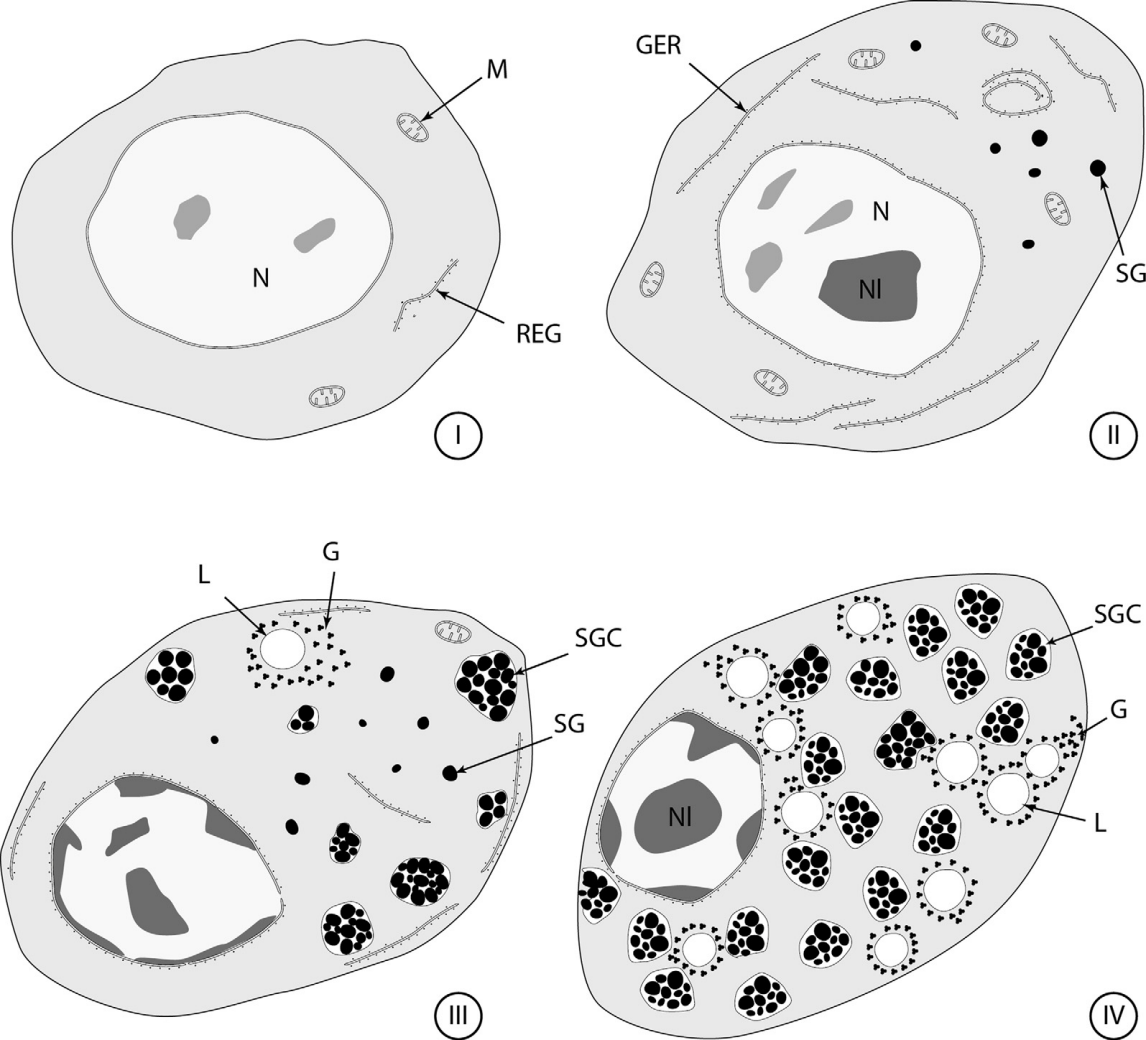
Diagram showing the four stages (I–IV) of vitellogenesis in *Crepidostomum metoecus*. G; glycogen particle; GER: granular endoplasmic reticulum; L: lipid droplet; M: mitochondrion; N: nucleus; Nl: nucleolus; SG: shell globule; SGC: shell globule cluster.

**Figure 16.**
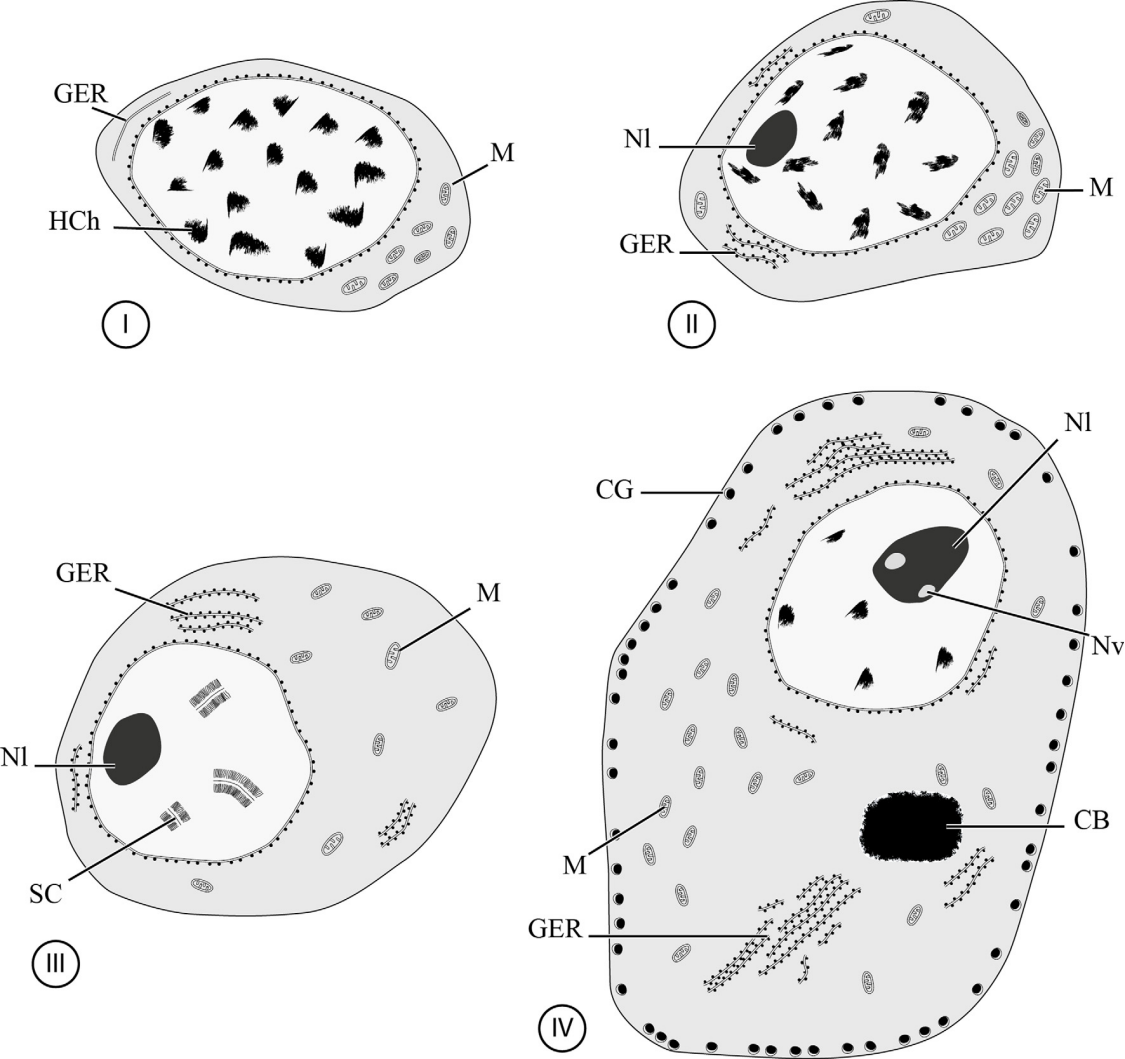
Diagram showing the four stages (I–IV) of the oogenesis of *Crepidostomum metoecus*. CB: chromatoid body; CG: cortical granule; GER: granular endoplasmic reticulum; Hch: heterochromatin; M: mitochondrion; Nl: nucleolus; Nv: nuclear vacuole; SC: synaptonemal complex.

## Discussion

### Vitellogenesis

Although vitellogenesis has a similar evolution in all neodermatan trematodes, some ultrastructural variations occur during maturation. Some vitelline follicles of Digenea possess interstitial cells (or nurse cells) with cytoplasmic extensions between vitellocytes that are believed to be involved in the selection and transport of materials from the parenchyma to the developing vitelline cells [1, 13, 14, 28]. In *Crepidostomum metoecus*, as in *Plagiorchis elegans*, or *Maritrema feliui*, nurse cells were not found [15, 46]. Vitelline cells provide nutrition for the eggs, and also provide the eggshell substance, in addition to the Mehlis’ gland [13, 28]. The abundance of endoplasmic reticulum, Golgi complexes, and free ribosomes, produces this high synthetic activity [24]. This synthesis gives rise to shell globules that coalesce to form clusters of dense globules surrounded by a limiting membrane (polygranular content). The shape and amount of shell globules in the cluster, the number of clusters per cell, and the size of shell globules and clusters, differ by species (Table 1). Data listed in this table were either taken from the text of corresponding publications or obtained by measuring the structures from figures in these publications [15]. Vitelline cells accumulate nutritive reserves (lipid droplets and glycogen particles) for the developing embryo [30]. The nutritive amount varies among digenean lineages (Table 1). Thus two groups of trematodes are observed: those whose vitelline cells produce a large amount of nutritive reserves as is the case in *C. metoecus* [7, 8, 15], and those whose vitellocytes contain a small amount of nutritive substances [22, 23]. Poddubnaya et al. (2013) investigated the vitellogenesis of *Brandesia turgida* and showed that the cytoarchitecture of vitellocytes reveals a specific life cycle. According to their analysis, the development of *C. metoecus* larva seems to occur after egg-laying, due to the high quantity of nutritive reserves in vitellocytes, unlike *Brandesia turgida*, which possess few nutritive substances [38]. The presence of such substances in eggs is necessary for the developing embryo that is less able or not able to receive nutrients from the parent worm. In vitelline cells of *C. metoecus*, only β-glycogen particles have been observed, as in other species [24, 30, 37, 42]. In some trematodes, α-glycogen rosettes and β-glycogen particles have been found [15]. The lack of densely coiled endoplasmic reticulum in fully mature vitellocytes of *C. metoecus* contributes to the conclusion that maturation ended at the fourth stage. In some other digenean species, a fifth stage presented secretion of densely coiled endoplasmic reticulum [12, 15, 30] and glycogen particles that are condensed in the whole cytoplasm. Moreover, vitelline cells providing shell globules participate in eggshell formation to protect the developed embryo. In the present study, we observed a great amount of shell globule clusters (about 30 per cell cross-section) composed of many shell globules (>30 globules/cluster). Nevertheless, the cluster size is one of the lowest in the vitelline gland of digenean species (Table 1). The variation of eggshell supply can be explained by the difference of life cycle between trematodes [1]. This feature can represent a phylogenetic point of comparison between families of trematodes, like many other characteristics.

**Table 1.** Ultrastructural characteristics of mature vitellocytes of *Crepidostomum metoecus* compared with those of other studied trematodes.

Families	Species	Cell size	L	L size	G	CER	SG/SGC	SGC	SGC size	References
Allocreadiidae	*Crepidostomum metoecus*	19	10	1.4	+	−	32	30	1.8	Present study
Azygiidae	*Azygia lucii*	15	5	1.5	+	−	50	19	2.7	[37]
Cryptogonimidae	*Aphallus tubarium*	7	1	2	+	+	35	3	2	[13]
	*Metadena depressa*	8	2	1	+	−	45	4	3	[14]
Derogenidae	*Halipegus eccentricus*	10	0	0	−	−	60	12	2	[22]
Dicrocoeliidae	*Dicrocoelium dendriticum*	18	1	1.5	+	+	42	12	3	[30]
Diplostomidae	*Pharyngostomoides procyonis*	/	1	1	/	+	13	14	1.3	[30]
Fasciolidae	*Fasciola hepatica*	20	0	0	+	+	25	6	2	[24]
Gorgoderidae	*Gorgoderina vitelliloba*	7	1	1.5	−	−	95	5	2	[23]
	*Phyllodistomum angulatum*	10	3	2.5	+	−	100	11	2.7	[37]
Haploporidae	*Haploporus lateralis*	/	1	/	+	−	/	/	/	[42]
Microphallidae	*Maritrema feliui*	16	1	1	+	−	73	5	2	[46]
	*Maritrema linguilla*	10	4	1	+	/	/	11	3	[21]
Plagiorchiidae	*Plagiorchis elegans*	20	13	2	+	+	30	20	2	[15]
	*Brandesia turgida*	13	1	1	−	+	25	/	2.5	[38]
Schistosomatidae	*Schistosoma mansoni*	13	14	1	+	+	27	14	2	[7, 8]

CER: coiled endoplasmic reticulum; G: glycogen; L: lipid droplets; SG/SGC: number of globules per cluster; SGC: shell globule clusters; (+/−): presence/absence of considered character; (/): no data on the considered character. Families are given in alphabetical order. Sizes are in μm. Data are averages of measurements on 10 cells.

### Oogenesis

On the basis of the female gonad structure, Platyhelminthes are subdivided into two levels of organization. In Neoophoran Platyhelminthes, the production of yolk and shell-forming precursors occurs in vitellaria, unlike in Archoophorans where it takes place in germaria [9]. The presence of organelles between growing oocytes supposes the presence of interstitial cells that may transport organelles. The initial phase of oocyte maturation takes place during the prophase of the first meiotic division in the ovary. As for *Metadena depressa* [14], no nucleus has been observed in the thin interstitial cytoplasmic layer between growing oocytes and the basal lamina, unlike in other Platyhelminthes where syncytial structures may occur in the ovary [34]. Oogonia are present along the wall of the ovary as for *Cryptocotyle lingua* [5], but do not contain a nucleolus-like *Zygocotyle lunata* [49]. Developing oocytes enter the zygotene-pachytene stage of the first meiotic division recognizable by the presence of synaptonemal complexes in the nucleoplasm, except in some digenean species where it takes place in the uterus [18]. During maturation, oocytes migrate to the center of the ovary. The metaphase takes place in the proximal part of the uterus where fertilization occurs [17]. A granular mass transfer from nucleus to cytoplasm occurs before the last stages of maturation. This material, named either “chromatoid body”, “nuclear extrusion”, or “nucleolus-like cytoplasmic body” [4, 18, 27, 35], probably contains RNA [4, 25]. This structure is often surrounded by mitochondria and granular endoplasmic reticulum [14], as in the present study. Movements of mitochondria during oogenesis were described by Yosufzai (1952), who studied this in *Fasciola hepatica*, where mitochondria are at one pole of oogonia, then form a ring around the nucleus in growing oocytes, and are finally evenly distributed in the cytoplasm of mature oocytes [52]. Such movement has not been observed in *C. metoecus*. In the fully mature oocytes, inclusions are obvious near the cytoplasmic membrane. These small vesicles initially randomly distributed in the cytoplasm, migrate to the cortical cytoplasm where they form a monolayer just beneath the oolemma. This can be observed in several fully mature digenean oocytes [2, 4, 6, 11, 18, 19, 27, 31, 34, 36, 49, 51]. The function of these granules could be the same as that of oocytes of other animals, which act in the blocking of polyspermy during fertilization [31]. The Thiéry method [47] allows us to exclude protein composition of these particles. The low protein composition of mature oocytes differs from that of other studied trematodes, such as *Paramphistomum cervi* [20], *Gastrothylax crumenifer*, and *Ceylonocotyle dawesi* [26]. This pattern, associated with the large nutrient content of vitellocytes of *C. metoecus*, enables us to consider that oocytes do not take part in the nutrition of the future embryo of the miracidium. Although some minimal differences occur between oogenesis of *C. metoecus* and some other digeneans, the general pattern of oogenesis corresponds to other neodermatan worms, and more generally to Platyhelminthes.
